# Comparative Genomics of *Lactobacillus crispatus* from the Gut and Vagina Reveals Genetic Diversity and Lifestyle Adaptation

**DOI:** 10.3390/genes11040360

**Published:** 2020-03-27

**Authors:** Qiuxiang Zhang, Lili Zhang, Paul Ross, Jianxin Zhao, Hao Zhang, Wei Chen

**Affiliations:** 1 State Key Laboratory of Food Science and Technology, Jiangnan University, Wuxi 214122, China; zhangqx@jiangnan.edu.cn (Q.Z.); 17851318862@163.com (L.Z.); zhaojianxin@jiangnan.edu.cn (J.Z.); zhanghao61@jiangnan.edu.cn (H.Z.); 2 School of Food Science and Technology, Jiangnan University, Wuxi 214122, China; 3 International Joint Research Center for Probiotics & Gut Health, Jiangnan University, Wuxi 214122, China; 4 APC Microbiome Ireland, University College Cork, Cork, Ireland; 5 National Engineering Research Center for Functional Food, Jiangnan University, Wuxi 214122, China; 6 Wuxi Translational Medicine Research Center and Jiangsu Translational Medicine Research Institute Wuxi Branch, Wuxi 214122, China; 7 Beijing Innovation Centre of Food Nutrition and Human Health, Beijing Technology and Business University (BTBU), Beijing 100048, China

**Keywords:** *Lactobacillus crispatus*, comparative genomics, evolution, host adaptation, acid tolerance

## Abstract

*Lactobacillus crispatus* colonizes the human feces, human vagina, and the crops and ceca of chicken. To explore the genetic characteristics and evolutionary relationships of *L. crispatus* isolated from different niches, we selected 37 strains isolated from the human vagina (n = 17), human feces (n = 11), and chicken feces (n = 9), and used comparative genomics to explore the genetic information of *L. crispatus* from the feces and vagina. No significant difference was found in the three sources of genomic features such as genome size, GC content, and number of protein coding sequences (CDS). However, in a phylogenetic tree constructed based on core genes, vagina-derived *L. crispatus* and feces-derived strains were each clustered separately. Therefore, the niche exerted an important impact on the evolution of *L. crispatus*. According to gene annotation, the *L. crispatus* derived from the vagina possessed a high abundance of genes related to acid tolerance, redox reactions, pullulanase, and carbohydrate-binding modules (CBMs). These genes helped *L. crispatus* to better adapt to the acidic environment of the vagina and obtain more nutrients, maintaining its dominance in the vagina in competition with other strains. In feces-derived bacteria, more genes encoding CRISPR/Cas system, glycoside hydrolases (GHs) family, and tetracycline/lincomycin resistance genes were found to adapt to the complex intestinal environment. This study highlights the evolutionary relationship of *L. crispatus* strains isolated from the vagina and feces, and the adaptation of *L. crispatus* to the host environment.

## 1. Introduction

*Lactobacillus crispatus* is an obligately homofermentative, catalase-negative, Gram-positive bacterium [1]. Numerous studies have shown the considerable probiotic potential of *L. crispatus*, such as modulating the host’s immune system [2], reducing allergic symptoms in mice [3], down-regulating the expression of cancer genes, and inhibiting the proliferation of MDA-MB-231 breast cancer cell lines [4]. In addition, as one of the most detected *Lactobacillus* species in the healthy female vagina [5], *L. crispatus* can inhibit vaginal pathogenic microorganisms, and maintain the health of the female reproductive system [6,7,8]. Moreover, clinical trials have shown the therapeutic effect of *L. crispatus* on female vaginitis and recurrent urinary tract infection [9,10].

In recent years, with the rapid development of gene sequencing technology, we can conduct in-depth researches on the genetic and functional diversity of bacteria at the genome level. Comparative genomics provides favorable support for understanding the basic genetic features, species evolution, and host adaptation of strains [11,12]. Teija Ojala et al. compared the pan- and core genomes of 10 *L. crispatus* strains, and revealed that some core genes implicated in protecting the urogenital tract from *Gardnerella vaginalis* colonization [13]. Van der Veer et al. found that glycosyltransferase (GT) genes were more abundant in *L. crispatus* isolated from vaginal samples with dysbiotic vaginal microbiota than that from the vaginal tracts of women with *Lactobacillus*-dominated vaginal microbiota (LVM). The vaginal niche in a dysbiotic state was under some immune pressure. The GTs regulated the surface glycogen of *L. crispatus* through a phase change, helping it escape immunity [14].

Other species of *Lactobacillus* that have been subjected to comparative genomic analysis are *L. reuteri* [15], *L. salivarius* [16], *L. curvatus* [17], and *L. brevis* [18]. The results showed that niches affected the genetic characteristics and evolutionary orientations of the given species. Meanwhile, the strains exhibited a specific host adaptability during the evolution process. However, the above studies only compared *L. crispatus* isolated from the same niche (vagina). Therefore, the differences in the genetic information inherent in *L. crispatus* isolated from different niches remain unclear. Recently, comparative genomics of two *L. crispatus* strains from the vagina and feces showed that the two isolates differed slightly in terms of genomic size, exopolysaccharides, and CRISPR–Cas system [19]. However, this work did not reveal the genetic relationships of strains from different habitats, and only focused on limited number of strains.

In this study, we analyzed 37 *L. crispatus* strains isolated from the human vagina, human feces, and chicken feces. Comparative genomics was applied to analyze whether feces-derived strains and vagina-derived strains differed significantly in basic genetic features and evolutionary relationships. In addition, the genes of *L. crispatus* associated with intestinal or vaginal adaptation were highlighted.

## 2. Materials and Methods

### 2.1. L. crispatus Strain Isolation

The human and chicken feces, and the vaginal swabs from healthy people were plated on the *Lactobacillus*-selective medium (LBS) agar under anaerobic conditions for 48 h at 37 °C [20]. Colonies with morphology (white, round, central raised, irregular edges) similar to *L. crispatus* were picked and streaked on LBS agar. One single colony was chosen from each plate and amplified using *L. crispatus*-specific primers (forward: 5′-ATTGATCGGAAGCGCAGTCT-3′, reverse: 5′-CAGTTGGAGTGCGTGAAAGG-3′). PCR was performed as follows: 95 °C for 5 min, 34 cycles (95 °C for 30 s, 58 °C for 40 s, 72 °C for 40 s), and 72 °C for 5 min. The products were then subjected to 1% agarose gel electrophoresis at 120 V for 30 min. The strains with the target band were 16S-rRNA-sequenced to further identify the species. In total, 37 strains of *L. crispatus* were separated from different samples, including 17 strains from human vaginal samples, 11 from human feces, and 9 from chicken feces (Table 1). These strains were incubated in de Man, Rogosa, and Sharpe (MRS) broth for 24 h at 37 °C. After centrifugation at 8000 rpm for 5 min, the bacterial cells were washed three times with sterilized water and stored at −80 °C before sequencing. Genomic DNA was extracted using a bacterial DNA extraction kit (OMEGA, Norcross, GA, USA) according to the manufacturer’s instructions. The quality of the extracted genomic DNA was determined by agarose gel electrophoresis (1% gel concentration), and the purity of the DNA was examined using an ultra-micro ultraviolet spectrophotometer. The concentration of DNA was determined using a Qubit^TM^4 fluorometer and Qubit^®^ DNA Assay Kit (Life Technologies, USA).

The study (Ethical No. KS202006) was approved by the clinical new technology and scientific research ethics committee of the Wuxi People’s Hospital, Wuxi, Jiangsu province, China.

### 2.2. Genome Sequencing, Assembly, and Annotation

The draft genomes of *L. crispatus* were sequenced using Illumina Hiseq×10 platform (Majorbio BioTech Co, Shanghai, China). Each sample provided an amount of raw sequencing data with no less than 100× coverage depth of the genome. The genome sequences were assembled using the software SOAP denovo2 (v2.0) [21]. The protein-coding sequences (CDS) were predicted using Glimmer according to Delcher et al. [22]. The tRNAscan-SE (v2.0) [23] and Barrnap were used to predict the tRNA and rRNA of genomes, respectively. The CDS of predicted genes were annotated across the Kyoto Encyclopedia of Genes and Genomes (KEGG) database to obtain functional information [24].

### 2.3. Average Nucleotide Identity Calculation

The average nucleotide identity (ANI) value between any two genomes was calculated using ANI perl script. The resulting matrices were clustered and visualized using R-packages’ heat map software (v1.0.8).

### 2.4. Pangenome and Core Genome Analysis

The pangenome and core genome of the 37 *L. crispatus* genomes were calculated using the PGAP1.21 [25], and the pangenome model was analyzed according to Heap’s law.

### 2.5. Phylogenetic Analysis

The protein sequences of the 37 *L. crispatus* were analyzed by OrthoMCL1.4 [26]. The protein families with the same function were then clustered using the Markov cluster algorithm (MCL). Core genes and specific genes were shown in a Venn plot. The core genes were aligned using MAFFT-7.313 [27], clustering analysis was performed using the neighbor-joining (NJ) method [28], and phylogenetic trees were constructed using PHYLIP (v3.6).

### 2.6. Carbohydrate Active Enzyme Analysis

The strain’s carbohydrate metabolism genes were annotated using the Carbohydrate Active Enzyme Database (CAZy) [29].

### 2.7. Statistical Analysis

Difference between two groups was evaluated using an independent samples *t*-test (SPSS 19.0), and a *p* value of < 0.05 was considered to indicate a significant difference.

## 3. Results

### 3.1. General Genome Features of the L. crispatus Strains

**Figure 1:**
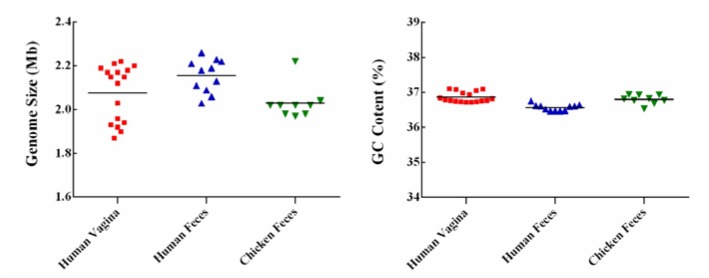
Genome size and GC content of *L. crispatus*. The red square, blue triangle, and green inverted triangle represent *L. crispatus* isolated from the human vagina, human feces, and chicken feces, respectively.

**Figure 2:**
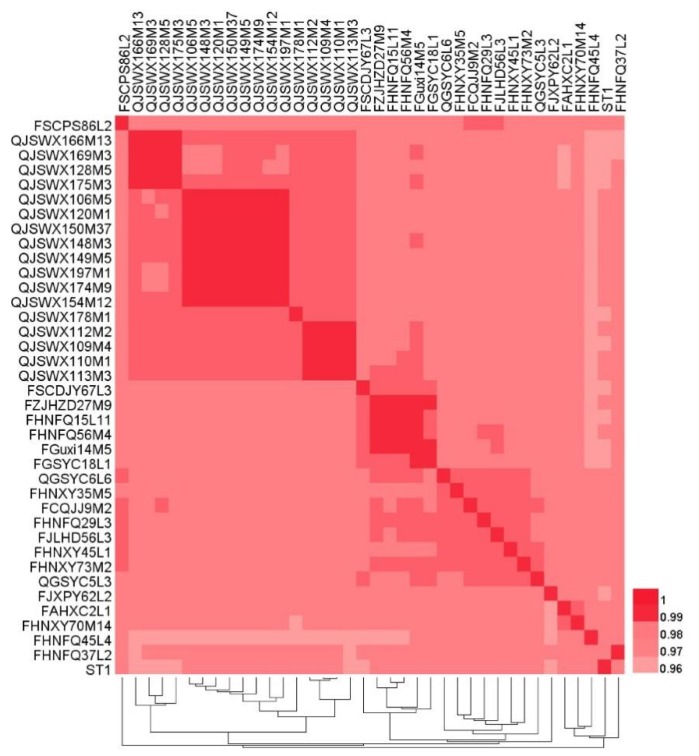
Heatmap showing the Average Nucleotide Identity (ANI) value among 38 *L. crispatus* strains.

The 37 *L. crispatus* genomes ranged in size from 1.87 Mb to 2.26 Mb, with an average length of 2.09 Mb (Figure 1). Genomic sizes of vaginal-source bacteria (range 1.87–2.22 Mb, ~2.08 Mb) were similar to those of strains isolated from chicken (range 1.97–2.22 Mb, ~2.03 Mb). The genomes of the human-fecal-origin *L. crispatus* (range 2.03–2.26 Mb, ~2.16 Mb) were the largest. The number of CDS varied from 1869 to 2325, with an average of 2110 (Table 1). The GC content of the genomes was on average 36.76%, and no significant difference was found among the three sources (Figure 1). The ANI value of the 37 strains compared with the reference strain *L. crispatus* ST1 from NCBI and between any two strains was 96.27% to 99.96% (Figure 2), i.e., greater than 96%, indicating that they belonged to the same species [30]. As shown in Figure 2, the genomes of vaginal *L. crispatus* strains clustered separately from the feces isolates based on hierarchal clustering of ANI values. However, the human fecal-origin *L. crispatus* did not form a separate cluster from the chicken isolates. The feces-derived strains had a closer genetic relationship.

### 3.2. Pan-genome and Core Genome Analysis of L. crispatus

**Figure 3:**
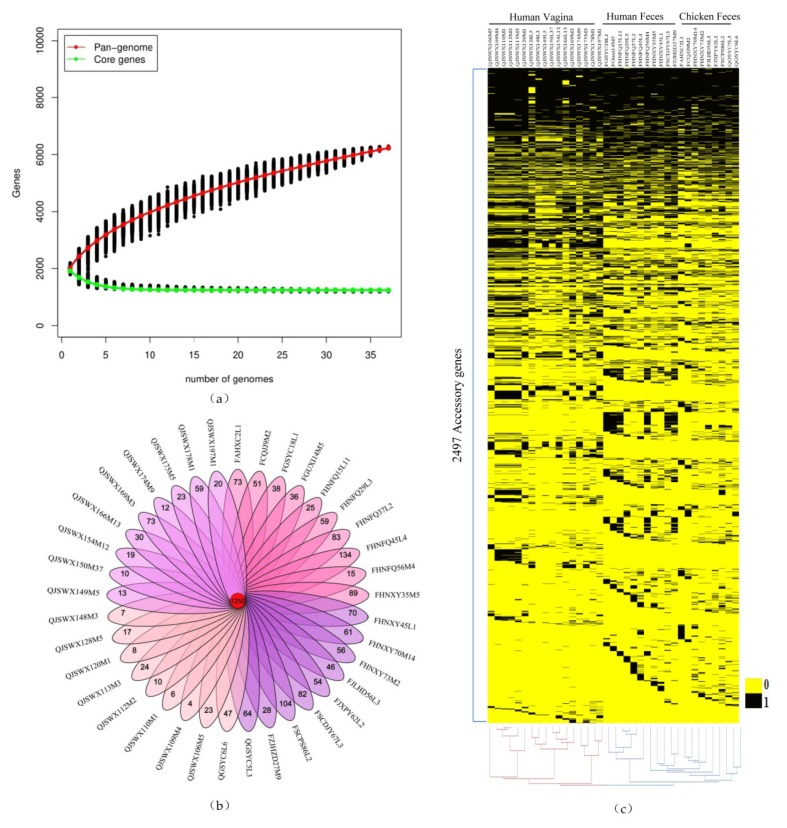
Pan-genome and core genome of *L. crispatus.* (**a**) The number of new genes accumulated in the *L. crispatus* pan-genome was plotted against the number of new genomes added and the number of accumulated genes attributed to the core genome relative to the number of added genomes. (**b**) Venn diagram representing the core and specific genes of 37 *L. crispatus* strains obtained by MCL clustering. The number in the middle red circle represents the number of core genes of the 37 strains. The numbers on the petals indicate the specific genes of each strain. (**c**) A heatmap representation of accessory genes against sequenced *L. crispatus* genomes. Each row represents an accessory gene. Each column represents a strain.

The pan-genome of 37 strains contained 5014 genes. As the number of strains increased, the pan-genome curve gradually converged. The derived mathematical function had an index value of less than 0.5, indicating that *L. crispatus* has a closed pan-genome [16]. However, the core genome only consisted of 1250 genes. The number of core genes was saturated and the gene curve trend was stable after the first 30 strains were analyzed (Figure 3a). The results showed that the 37 strains of *L. crispatus* in this study fully represented this species. According to the result of MCL clustering, the core genes, accessory genes, and specific genes of 37 strains were obtained. An accessory gene is defined as a gene shared by at least two strains. A specific gene is a gene that only exists in a certain strain. The number of specific genes in *L. crispatus* varied from 4 to 134 (Figure 3b). The average number of specific genes of *L. crispatus* isolated from chicken and human feces was 61 and 57 respectively. However, *L. crispatus* strains isolated from vaginal samples had an average number of specific genes of only 21.

Accessory genes contribute to species diversity. They are not important for bacterial growth, but confer selective advantages such as adaptation to different niches, drug resistance, or colonization of new hosts [31]. The distribution of the 2497 accessory genes was different in the 37 strains, as visualized by heat map (Figure 3c). It was shown that the 37 *L. crispatus* strains were divided into two large branches; one branch was vaginal-derived strains and the other was strains from feces. This indicated that the accessory genes were affected by the habitat. Meanwhile, strains isolated from chicken or human feces were similar in the composition of their accessory genes.

### 3.3. Phylogenetic Analysis of L. crispatus Strains

**Figure 4:**
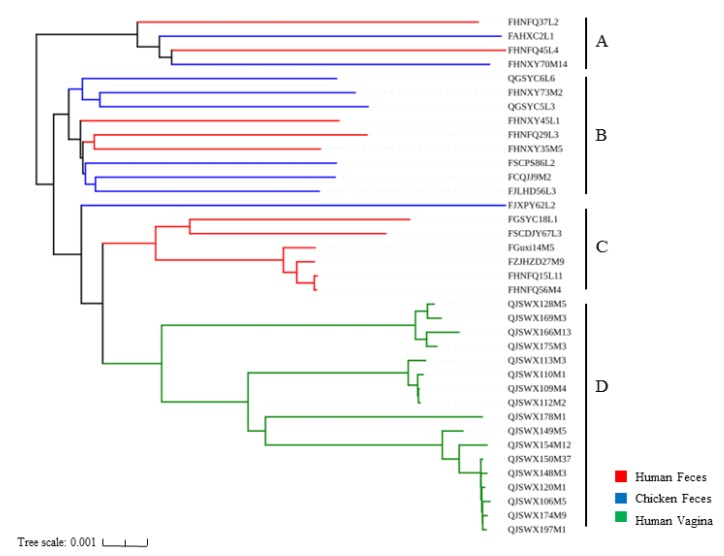
Phylogenetic tree of 37 *L. crispatus* strains. Based on 1091 single-copy core genes of 37 strains, the phylogenetic tree was constructed using the neighbor-joining method. According to the clustering results of the phylogenetic tree, the phylogenetic tree is divided into 4 branches, namely Branch A, B, C and D.

To explore the effects of different niches on the phylogeny of the *L. crispatus* genome, 1091 single-copy orthologous genes were used to construct a phylogenetic tree (Figure 4) against the 37 strains via the neighbor-joining method. The feces-derived strains and the vagina-derived strains were each clustered into different clusters. The phylogenetic tree was divided into four branches, including Branches A, B, C, and D. All 17 vagina-isolated strains were clustered into Branch D. However, chicken and human isolates were distributed in either Branch A or Branch B. Branch C contained only six *L. crispatus* strains, all from human feces. Therefore, different habitats might relate to differences in the genetic evolution of the strains.

### 3.4. Evolution and Adaptation to Environment

**Figure 5:**
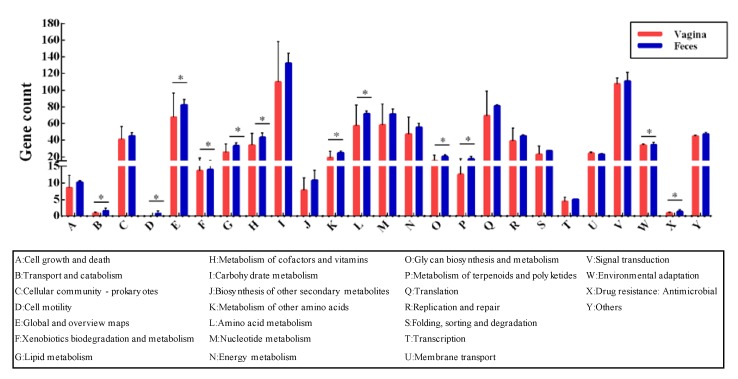
Annotation for genes of *L. crispatus* isolated from vagina and gut using the KEGG database. * *p* < 0.05.

To investigate whether *L. crispatus* showed some adaptability to different habitats in terms of gene function, we performed KEGG annotation for all 37 strains. However, based on the results of ANI value, phylogenetic tree, and the distribution of the accessory genes, we found that the feces-derived (human/chicken) strains had a high degree of similarity. Therefore, in terms of subsequent habitat adaptation analysis, we mainly focused on the adaptation of fecal-derived strains and vagina-derived strains to their respective niches. Across the two niches, genes associated with carbohydrate metabolism (~13%) and membrane transport (~11%) occupied a large proportion. The genes encoding transport and catabolism, cell motility, global and overview maps, xenobiotics biodegradation and metabolism, lipid metabolism, metabolism of cofactors and vitamins, metabolism of other amino acids, amino acid metabolism, glycan biosynthesis and metabolism, metabolism of terpenoids and polyketides, and drug resistance were annotated. Significant differences (*p* < 0.05) were observed between the two niches in genes involved with antimicrobial and environmental adaptation (Figure 5).

To further explore genes that might be adaptive to the vaginal and intestinal environment, we analyzed the genes with significant differences (*p* < 0.05) in gene abundance of each KEGG orthology between the two sources. Meanwhile, if a gene was present in more than half of the fecal strains, but in fewer than half in the vaginal strains, this gene was considered adapted to the gut. The genes associated with vaginal adaptation were also analyzed according to the same method. Based on this criterion, 69 important genes were found. Among the feces-derived *L. crispatus* isolates, 31 genes were analyzed, including 11 fecal-specific genes, (i.e., only present in feces). Of 38 genes found in the vagina-derived strains, 7 were vagina-specific genes.

Of the 31 genes in the feces-derived strains, 22% were related to the CRISPR–Cas system, and 19% to carbohydrate metabolism (deoxyribose-phosphate aldolase, sucrose phosphorylase, α-galactosidase). Other genes were linked to biosynthesis of other secondary metabolites and to amino acid metabolism. In the 38 genes of vagina-derived *L. crispatus*, 31% were related to carbohydrate metabolism (galactitol-specific IIC component, pullulanase, hexulose-6-phosphate isomerase). In addition, there were many genes involved in redox reactions and acid tolerance in the vagina, such as iron–sulfur protein and manganese ion transporters (Tables S1 and S2).

### 3.5. Active Carbohydrate Enzymes

The carbohydrate-utilization-related genes occupied the largest proportion in each genome by KEGG annotation. Therefore, we further compared the number of genes encoding carbohydrate-active enzymes in strains from feces and vagina.

**Figure 6:**
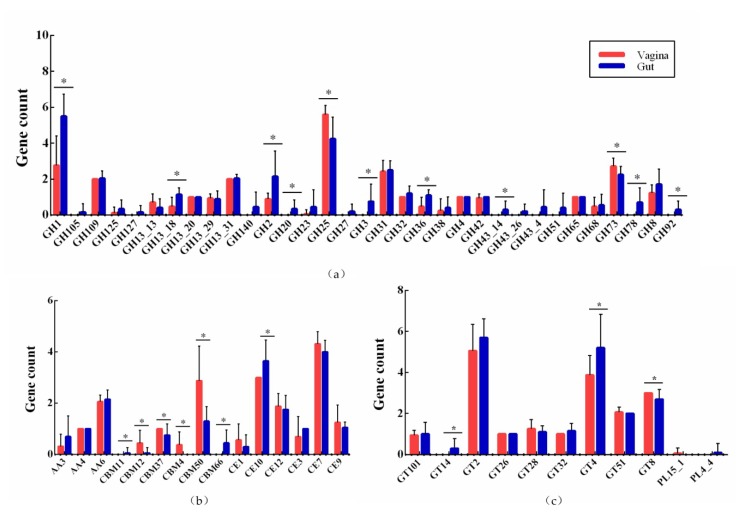
Distribution and abundance of active carbohydrate enzyme family genes in *L. crispatus* strains isolated from feces and vagina. * *p* < 0.05.

The 37 strains had genes encoding 33 glycoside hydrolases (GHs) families. The GH family was the most important type of carbohydrate enzyme in *L. crispatus*, accounting for 55.9%. The abundance of GH1, GH13_18, GH2, GH20, GH25, GH3, GH36, GH43_14, GH73, GH78, and GH92 differed significantly (*p* < 0.05) between strains isolated from the feces and vagina. *L. crispatus* strains of fecal origin contained more types of carbohydrate enzyme than did *L. crispatus* of vaginal origin. Twelve GH families were only present in strains derived from feces. The GH140 and GH43_4 only existed in human feces isolates, whereas GH105 were only present in strains isolated from chicken feces (Figure 6a).

Additionally, nine glycosyl transferases (GTs), three auxiliary activities (AAs), six carbohydrate-binding modules (CBMs), six carbohydrate esterases (CEs), and two polysaccharide lyases (PL) were detected in 37 strains. The abundances of GT14, GT4, GT8, CE10, and CBM families were significantly different between the two groups. The CBM4 and PL15_1 were present only in vaginal strains (Figure 6b,c).

## 4. Discussion

Comparative genomic analysis of 37 *L. crispatus* strains from fecal and vaginal sources revealed the host’s influence on the genetic characteristics and evolution of the strain, as well as the adaptability of the strain to the intestine and vagina.

The general genomic characteristics in terms of size, GC content, and number of CDS (Table 1) showed that there was no significant difference between the *L. crispatus* strains derived from the three sources. However, when compared with chicken feces and human vaginal isolates, the genome size of strains isolated from human feces was larger. A possible explanation is that the human gut placed more environmental pressure on the bacteria [32]. To survive in a complicated environment, the *L. crispatus* could have obtained additional genes through horizontal transfer [33].

The phylogenetic tree results showed that the fecal isolates and the vaginal isolates were each clustered separately (Figure 4). This distinction indicated that the niche exerted an important impact on the evolution of the *L. crispatus* [15]. This result provided the insight that we might be able to determine the niche of an unknown source *L. crispatus* based on the clustering results of strains in the phylogenetic tree. A previous study revealed that Bifidobacteria isolated from the vagina and feces had no tendency to host in the phylogenetic tree [34]. In this case, it might have been that Bifidobacteria were not the main strain in the vagina. Therefore, the effect of the source on the evolutionary relationship of Bifidobacteria was not obvious.

Compared with the gut, the vagina has a lower pH (4 ± 0.5) [35]. Low pH helps reduce the risk of gynecological diseases and maintain female reproductive health [36]. Therefore, *L. crispatus* from the vagina require more defense mechanisms to respond to the low pH. The gene encoding manganese transport protein was presented only in vagina-sourced strains. This protein can take up Mn^2+^ and expel protons out of the cell to take part in the acid response, maintaining intracellular pH homeostasis [35]. Meanwhile, Mn^2+^ can also help in protecting bacteria against oxidative stress [37].

Lactobacilli in the vagina produce high levels of hydrogen peroxide [38,39]. Hydrogen peroxide is an important antibacterial substance. It can inhibit such pathogens as *Gardnerella vaginalis* and *Neisseria gonorrhoeae* in the vagina, and help maintain the normal flora of the vagina [40]. Meanwhile, hydrogen peroxide also causes oxidative stress in *L. crispatus*. Therefore, we observed that genes associated with oxidative stress were significantly enriched in vaginally derived bacteria, as described in Table S1. The two-component system is a broad signal transduction pathway in bacteria. It plays a major role in adapting to changing environmental conditions such as cell envelope stress response, phosphate regulation, and oxidative stress [37,41]. The OmpR family significantly increases the resistance of bacteria to hydrogen peroxide [42]. The genes encoding Fe-S cluster assembly protein were more abundant in the vagina (Table S1), and these genes are critical in the catalysis of electron transfer or metabolic support reactions [43]. Meanwhile, the Suf protein complex helps to assemble or repair oxygen-labile Fe-S clusters under oxidative stress [44,45]. In addition, hydrogen peroxide could further react with some Fe^2+^ iron to result in high-activity oxidants through the Fenton reaction [46]. This highly active oxidant might have a stronger inhibitory effect on pathogens in the vagina and a more positive effect on reproductive health, but this requires further testing to verify.

Pullulanase is an enzyme that breaks down glycogen [14]. In the vagina, glycogen is the main source of carbohydrates for microorganisms [47]. Genes encoding pullulanase were more abundant in the vagina-derived *L. crispatus* strains than in strains of fecal origin (Table S1). *L. crispatus* uses this enzyme to break down glycogen and produce lactic acid, which could maintain the low pH and inhibit pathogenic bacteria in the vagina [48]. The genes coding clumping factor A and A GntR family transcription factor were only present in the vagina-derived *L. crispatus* strains (Table S1).

Due to the variety and high density of bacteria, the intestine is an ideal niche for phage survival [49]. The *L. crispatus* isolated from feces were more susceptible to phage infection. The CRISPR/Cas system is known as the prokaryotic immune system and resists the invasion of foreign genetic material such as phage viruses and foreign plasmids [50]. To protect against damage to cells by foreign DNA, more genes related to the CRISPR–Cas system were observed in feces-derived *L. crispatus* (Table S2).

Tetracycline and lincomycin resistance genes were present only in the fecal strains (Table S2). Antibiotics have been used extensively to treat diseases caused by bacterial infections in humans and animals. Correspondingly, bacteria have acquired certain resistance genes through horizontal transfer to improve antibiotic resistance and their own survival ability [51]. Gene transfer at the level of drug-resistance genes often occurs in the human gastrointestinal tract [52]. In addition, genes related to streptomycin biosynthesis, such as rfbA, rfbB, and rfbC, were significantly enriched in the feces-derived bacteria (Table S2). Meanwhile, some genes associated with galactose metabolism, cysteine and methionine metabolism, and arginine biosynthesis were found only in the fecal strains.

To increase our understanding of the ability of *L. crispatus* to use host-derived glycogen fermentation, the genomes of 37 *L. crispatus* were analyzed and compared using CAZymes. The feces-derived strain was enriched in various GH family enzymes, including GH140, GH20, GH3, GH43_14, GH78, and GH92, which are involved in the utilization of a variety of carbon sources (Figure 6a). This might be because the gut contains more types of carbohydrate than the vagina. *L. crispatus* would need more enzymes to break down these carbohydrates to provide them with energy. The abundance of GH2 (β-galactosidase) and GH 36 (α-galactosidase) isolates in human feces was significantly higher than human vagina and chicken feces. Milk and other dairy products are an important part of human diet. GH2 and GH36 can hydrolyze galactose in dairy products, which is beneficial to the growth of strains in the intestine [53]. However, the abundance of CBM12, CBM37, CBM4, CBM66, GH25, and GH73 family in the vagina was higher than in the gut. CBM is a non-catalytic member of the cellulolytic enzymes. It is thought to assist in the synergy of enzymes through proximity and targeting, and plays an important role in the degradation of insoluble substrates [54,55]. Cellulose is the main component of plant cell walls. Humans and chickens consume cellulose-containing vegetables, but the feces isolates had a lower CBM abundance than the vagina (Figure 6b). This might require more in-depth exploration of the specific role of CBM in vagina-derived *L. crispatus*. GH25 has a role in facilitating cell division and in defense, as a lysozyme involved in cell wall and peptidoglycan catabolism [56]. GH73 encodes β-N-acetylglucosaminidases that cleave the β-1,4 glycosidic linkage between the N-acetylglucosamine (GlcNAc) and N-acetylmuramic acid (MurNAc) residues of bacterial cell wall peptidoglycan (PG) [57]. These two GH family lysozymes might provide a bacterial strategy to improve the competitive advantage of *L. crispatus* by destroying the cell walls of other pathogenic bacteria in the vagina and lysing them [58]. In summary, the above results demonstrate that *L. crispatus* is adaptable to different environments to improve its ability to survive.

## 5. Conclusions

In this study, we selected the genomes of 37 *L. crispatus* strains isolated from the human vagina, human feces, and chicken feces. The results of comparative genomics demonstrated that niche affected the evolution of *L. crispatus* to a large extent. Moreover, the *L. crispatus* strains showed adaptability to different environments to improve their own survival ability during the evolution of the strain.

## Figures and Tables

**Table 1:** General genome features of the 38 *L. crispatus* strains.

Strains	Source	Genome Size (Mb)	GC Content (%)	CDS No.	tRNA No.	rRNA No.	Accession No.
ST1	Chicken Feces	2.04	36.9	2023	64	12	SAMEA2272191
FGSYC18L1	Human Feces	2.18	36.47	2177	37	3	SAMN12869300
FGuxi14M5	Human Feces	2.19	36.47	2191	42	1	SAMN12869301
FHNFQ15L11	Human Feces	2.26	36.48	2241	44	3	SAMN12869302
FHNFQ29L3	Human Feces	2.03	36.75	2053	56	3	SAMN12869303
FHNFQ37L2	Human Feces	2.09	36.60	2091	45	1	SAMN12869304
FHNFQ45L4	Human Feces	2.13	36.64	2190	44	3	SAMN12869305
FHNFQ56M4	Human Feces	2.23	36.52	2218	40	3	SAMN12869306
FHNXY35M5	Human Feces	2.11	36.61	2121	40	3	SAMN12869307
FHNXY45L1	Human Feces	2.06	36.62	2066	35	0	SAMN12869308
FSCDJY67L3	Human Feces	2.21	36.61	2190	45	1	SAMN12869309
FZJHZD27M9	Human Feces	2.22	36.47	2243	41	1	SAMN12869310
FAHXC2L1	Chicken Feces	2.04	36.81	2023	46	4	SAMN12869311
FCQJJ9M2	Chicken Feces	1.97	36.93	1988	44	3	SAMN12869312
FHNXY70M14	Chicken Feces	1.98	36.92	1985	45	3	SAMN12869313
FHNXY73M2	Chicken Feces	2.02	36.69	1989	49	3	SAMN12869314
FJLHD56L3	Chicken Feces	2.02	36.77	1970	38	3	SAMN12869315
FJXPY62L2	Chicken Feces	2.02	36.92	1977	41	3	SAMN12869316
FSCPS86L2	Chicken Feces	2.22	36.53	2203	49	3	SAMN12869317
QGSYC5L3	Chicken Feces	2.02	36.82	2003	45	3	SAMN12869318
QGSYC6L6	Chicken Feces	1.98	36.77	2004	43	3	SAMN12869319
QJSWX106M5	Human Vagina	2.15	36.82	2194	72	4	SAMN12869320
QJSWX109M4	Human Vagina	2.18	36.72	2231	53	3	SAMN12869321
QJSWX110M1	Human Vagina	2.17	36.72	2227	41	4	SAMN12869322
QJSWX112M2	Human Vagina	2.17	36.73	2241	53	3	SAMN12869323
QJSWX113M3	Human Vagina	2.22	36.77	2309	46	3	SAMN12869324
QJSWX120M1	Human Vagina	2.15	36.77	2206	68	4	SAMN12869325
QJSWX128M5	Human Vagina	1.94	36.73	1972	41	0	SAMN12869326
QJSWX148M3	Human Vagina	1.87	37.05	1869	43	2	SAMN12869327
QJSWX149M5	Human Vagina	1.96	37.08	1981	53	3	SAMN12869328
QJSWX150M37	Human Vagina	1.90	36.98	1914	40	3	SAMN12869329
QJSWX154M12	Human Vagina	2.03	37.1	2108	42	2	SAMN12869330
QJSWX166M13	Human Vagina	1.92	36.75	1962	42	1	SAMN12869331
QJSWX169M3	Human Vagina	2.21	36.74	2325	66	7	SAMN12869332
QJSWX174M9	Human Vagina	1.93	37.09	1969	49	3	SAMN12869333
QJSWX175M3	Human Vagina	2.12	36.79	2112	50	2	SAMN12869334
QJSWX178M1	Human Vagina	2.19	36.84	2243	71	6	SAMN12869335
QJSWX197M1	Human Vagina	2.20	36.94	2275	43	3	SAMN12869336

## Supplementary Materials

The following are available online at https://www.mdpi.com/2073-4425/11/4/360/s1, Table S1: Higher abundance of 38 genes in the vagina, Table S2: Higher abundance of 31 genes in the feces.
